# Modifiable factors which predict children’s gross motor competence: a prospective cohort study

**DOI:** 10.1186/s12966-019-0888-0

**Published:** 2019-12-11

**Authors:** Lisa M. Barnett, Jill A. Hnatiuk, Jo Salmon, Kylie D. Hesketh

**Affiliations:** 1 Institute of Physical Activity and Nutrition, Deakin University, Geelong, 3125 Australia; 2 School of Health and Social Development, Deakin University, Geelong, 3125 Australia; 3 School of Exercise and Nutrition Sciences, Deakin University, Geelong, 3125 Australia

**Keywords:** Early childhood, Physical activity, Object control, Locomotor, Home affordances

## Abstract

**Background:**

Fundamental motor skills (FMS) are important for physical activity and healthy weight status in children, yet it is unclear which early childhood factors facilitate subsequent motor skill. The aim of this prospective study was to investigate which modifiable family and home environment factors in the early years predict children’s FMS at age five.

**Methods:**

Mothers from the Melbourne InFANT program (registered with the International Standard Randomised Controlled Trial Number Register (ISRCTN81847050)) completed questionnaires when child was aged 4, 9, 19 months old, and 3.5 years old on factors hypothesised to predict motor skills. Some factors were grouped in tertiles (high, medium, low) due to the nature of the distribution. At 5 years old children were assessed on 6 locomotor and 6 object control skills (Test of Gross Motor Development-2). Eight regression models examined the association between factors at each time-point and children’s skills (object control and locomotor) at 5 years old.

**Results:**

The sample varied by time-point (178 to 259 children). Maternal physical activity optimism (4 months; β = 2.43), home physical activity equipment (9 months; β = 0.82), time outdoors – middle (9 months; β = 2.50) and highest tertile (9 months; β = 2.86), time free to move about - highest tertile (19 months; β = 2.41), time with older children - middle (19 months; β = 3.15) and highest tertile (3.5 years; β = 3.00) were predictive of better locomotor scores. Mothers’ own physical activity (9 months; β = − 0.01) and time active with mum – highest tertile (3.5 years; β = − 3.73) were negatively associated with locomotor skill. Time with older children - highest (4 months; β = 2.27) and middle tertile (19 months; β = 2.97), time free to move about – middle (19 months; β = 2.55) and highest tertile (19 months; β = 2.47), and more home equipment (9 months; β = 0.83); (3.5 years; β = 0.17) were predictive of better object control skills. Maternal physical activity knowledge (3.5 years; β = − 3.05) was negatively associated with object control skill.

**Conclusions:**

Providing a supportive environment with older children and equipment, and allowing toddlers’ freedom to move, appears important. Opportunities exist to educate parents on their important role in developing children’s motor skills. Clinicians could advise parents that the home environment can make a difference to their child’s FMS starting from infancy.

## Background

Motor skill competence (e.g. fundamental motor skills such as balancing, jumping, and throwing) is integral to children being physically active, thus reducing chances of developing unhealthy physical activity and weight trajectories [1, 2]. Motor competence is developing during the early years, the period up until 5 years old, and fundamental motor skills become the precursors to more specialised movement and sport skills. Despite the importance of fundamental motor skills to children’s health and development, research suggests children have poor motor competence. In Australia, only around half of children master skills such as throwing, kicking and jumping by the end of primary school (around age 12 years) [3], and this is reflective of data from other countries [4, 5].

Systematic review and meta-analysis evidence suggests that it is possible to improve fundamental motor skills, and also physical activity, through preschool teacher-led interventions [6]. Yet there is little understanding of what social and environmental factors are important to foster in the home environment so young children have the best chance of developing adequate fundamental motor skills. A recent review noted the dearth of research regarding social and environmental factors that might relate to children’s motor skills [7]. Furthermore, systematic reviews of motor skill correlates have only investigated potential correlates in children aged 3 and above [7, 8]. This is important to understand because children spend the majority of their time in the home environment during early life [9]. Parents (and clinicians who work with parents) have great potential to influence children’s motor skill development by creating environmental movement opportunities during the early years.

Cross-sectional studies in infant children indicate the home environment is important, with availability of toys (both fine and gross motor) considered to enhance motor development associated with motor development scores in Brazilian [10] and Japanese children [11]. Similarly, in another Brazilian study, home factors (physical space, daily activities, play materials) and global motor score at 9 months old were positively associated [12]. In 18-month old African children, more developmental stimulation, measured in terms of variety in play materials and activities where the child was engaged with an adult in the past 3 days, were associated with better motor scores [13].

In preschool aged children there is some relevant cross sectional research on correlates of motor skill. For example, in 2011, in a large sample of Belgium children, some evidence was found for family level correlates of motor skills [14]. In 2013, an Australian study reported more evidence for correlates of motor skill at the child level, than at the family and environmental levels [15]. More recently, in a disadvantaged sample from the United States, a range of child, family and environmental factors were associated with motor skills showing that correlates of fundamental motor skills are multidimensional [16].

There is a lack of longitudinal research focused on motor development pathways in children below school age (i.e. younger than 6 years) [17]. The literature has largely focused on motor milestones (rolling over, sitting up, crawling, and walking) as predictive factors for a range of health outcomes [18, 19], rather than parent modifiable practices and beliefs. One Brazilian cohort study assessed 49 infants three times over 4 months and found associations between home affordances, parental practices and knowledge, and infant motor development over this time [20].

Identification of modifiable social and environmental behaviours that could be undertaken by parents to enhance their children’s subsequent motor skills, could be used to inform movement-based guidelines for infants (until 12 months), toddlers (12–36 months) and pre-schoolers (typically aged 3–4 years) to assist parents, and clinicians who advise parents, such as early childhood nurses. Therefore, the purpose of this prospective study was to investigate which modifiable family and home environment factors in infancy, toddlerhood and the early preschool years predict children’s motor skills at age 5 years.

## Methods

This analysis used data from the Melbourne Infant Feeding, Activity and Nutrition Trial (InFANT) Program; which was a 15-month obesity prevention intervention (2008 to 2010) targeted to first-time parents and their infants between 4 and 19 months of age [21]. The original program aimed to educate and support first-time parents in their approach to their infant’s dietary, physical activity and sedentary behaviours. Parents were guided in development of their parenting skills regarding developmentally appropriate eating and physical activity behaviours in their infant. Post-intervention follow-up occurred when the child was aged 3·5 years (2011–2012) and 5 years (2013). Parents were recruited through parent groups conducted from child health centres (identified within randomly selected geographic government areas). A total of 542 parent–child pairs (86% response) from 62 different parent groups were involved in the original intervention, with 480 (89% retention) families enrolled at intervention end. The analyses in this paper does not report on intervention outcomes, but does account for intervention status in all models (as a precautionary measure, despite no differences in motor skill observed between groups; data not shown).

Data for the current study were drawn from five time points (when the child was aged: 4 months, 9 months, 19 months, 3·5 years and 5 years). Parents completed paper surveys when their child was aged 4 months, 9 months, 19 months and 3·5 years old. Children completed the motor skill assessments at 5 years only. Children were included in analyses if they had motor skill data and also data from the relevant time-point. Ethical approval to conduct the study has been granted by the University Ethics Committee and by the Victorian Office for Children. Parents gave written informed consent for themselves and their children.

Demographic information were collected at baseline via a written questionnaire from the responding parent (all mothers), including maternal country of birth (Australia/Other), highest level of maternal education (Low = Secondary school or less; Medium = Trade certificate/diploma; High = University qualification), maternal and child date of birth (to calculate age) and child sex. Age the child began crawling and walking were attained from maternal report questionnaires when child was aged 19 months.

The questionnaire described above also asked parents about a range of child and family factors investigated as potential correlates of motor skills. These included child behaviours (e.g., time spent on tummy), maternal beliefs (e.g., maternal self-efficacy), parental behaviours (e.g., maternal physical activity levels) and the home environment (e.g., number of equipment items available) relevant at each time-point. Table 1 describes the predictor variables at each time-point.

**Table 1:** Description of correlates included at each time point

Variable	Description and/or coding	Child aged 4 months Mean (SD) or tertile cut-points	Child aged 9 months Mean (SD) or tertile cut-points	Child aged 19 months Mean (SD) or tertile cut-points	Child aged 3.5 years^a^ Mean (SD) or tertile cut-points
**Child behaviours**					
Time spent being physically active with mum	Proxy-report of the amount of time in the last week. Due to distribution of the data, divided into tertiles (low, medium, high).	Low = 0–26 Med = 29–60 High = 64–428	Low = 0–34 Med = 39–86 High = 94–600	Low = 0–60 Med = 69–120 High = 150–360	Low = 0–420 Med = 435–480 High = 870–2820
Time spent having tummy time	Proxy-report of the amount of time in the last week. Due to distribution of the data, divided into tertiles (low, medium, high).	Low = 0–9 Med = 10–26 High = 30–257	N/A	N/A	N/A
Time spent on the floor (T1 & T2); Time spent ‘free to move around’ (T3 & T4)	Proxy-report of the amount of time in the last week. Due to distribution of the data, divided into tertiles (low, medium, high).	Low = 0–34 Med = 35–90 High = 94–428	Low = 4–120 Med = 129–240 High = 257–600	Low = 60–360 Med = 390–480 High = 510–1440	Low = 0–3360 Med = 3480–4620 High = 4680–5880
Time spent with other children of a similar age	Proxy-report of the amount of time in the last week. Due to distribution of the data, divided into tertiles (low, medium, high).	Low = 0–13 Med = 15–17 High = 19–480	Low = 0–17 Med = 20–34 High = 39–1371	Low = 0–30 Med = 60–90 High = 120–1440	Low = 0–600 Med = 630–1680 High = 1740–5400
Time spent with older children	Proxy-report of the amount of time in the last week. Due to distribution of the data, divided into tertiles (low, medium, high).	Low = 0–0 Med = 1–17 High = 26–343	Low = 0–9 Med = 17–26 High = 34–343	Low = 0–0 Med = 10–30 High = 60–600	Low = 0–0 Med = 50–420 High = 465–5580
Time spent outside	Proxy-report of the amount of time in the last week. Due to distribution of the data, divided into tertiles (low, medium, high).	Low = 0–17 Med = 19–43 High = 51–275	Low = 0–30 Med = 34–69 High = 77–257	Low = 0–60 Med = 90–120 High = 150–480	Low = 0–810 Med = 840–1260 High = 1320–2850
Organised activity	One item assessing children’s participation in an organised activity (Yes/No)	N/A	N/A	Yes = 49.0% No = 51.0%	Yes = 79.5% No = 20.5%
**Maternal beliefs**					
Maternal physical activity knowledge^b^	Composite score (scale 0–3) of ten^c^ questions examining the importance of physical activity for children’s health and development	2.51 (0.34)	N/A	2.54 (0.35)	2.46 (0.32)
Maternal physical activity views^b^	Composite score (scale 0–3) of four questions assessing mothers’ views of physically active children	0.88 (0.43)	N/A	0.73 (0.44)	1.85 (0.45)
Maternal physical activity optimism^b^	Composite score (scale 0–3) of 3 questions examining the anticipated ease of engaging children in physical activity	2.32 (0.44)	2.48 (0.47)	2.39 (0.45)	2.31 (0.51)
Maternal physical activity self-efficacy^b^	Composite score (scale 0–3) of 3 questions examining mothers’ confidence for promoting physical activity	2.49 (0.48)	2.57 (0.47)	2.51 (0.49)	2.36 (0.52)
Maternal floor concerns^b^	Composite score (scale 0–3) of two questions examining perceptions of safety of floor play	0.93 (0.54)	N/A	N/A	N/A
**Parental behaviours**					
Parental facilitation of physical activity	Summed score of questions on parental facilitation of physical activity for children (1 Never or rarely, 2 Some days each week, 3 Most days each week, 4 Every day, 5 At least once a day, 6 Several times each day), converted into weekly equivalent scores^d^	23.84 (5.76)	24.36 (5.70)	40.87 (12.75)	50.05 (10.12)
Maternal physical activity	Mothers self-reported using the Active Australia Survey. Reported in minutes per past week.	466.07 (355.78)	486.35 (368.42)	396.29 (375.29)	398.68 (340. 72)
**Home environment**					
Physical activity equipment in home	T1-T3: Number of age-appropriate equipment items for young children parents’ anticipate they will provide in their home^b^ T4: How frequently child uses age-appropriate equipment items in their home (1 Don’t have, 2 Never/rarely, 3 Less than once per week, 4 1–2 times per week, 5 3–4 times per week, 6 5–6 times per week, 7 Daily). Items at T4 were: Balls, bats/rackets, bike/ride on, climbing equipment, trampoline, swings/slides, swimming pool, skipping rope, scooter, other toys that encourage active play.	6.53 (2.16)	5.76 (1.18)	8.66 (1.90)	35.03 (9.27)

^a^T4 tertiles were capped as follows: time spent with other children/older children and time spent free to move about capped at 14 h per day (mean waking time for child of this age); time outdoors and time spent being physically active with mum capped at 7 h/day (half of waking day)

^b^Please refer to Hnatiuk et al., 2013 for further processing details

^c^At T4, two items within this scale were dropped as they were no longer relevant due to the age of the child

^d^This measure consisted of *n* = 2 items at T1 and T2, *n* = 6 items at T3 and *n* = 7 items at T4

The processing of many of these variables is consistent with previous research with this sample [22]. However, in this sample some items were grouped in tertiles (high, medium, low) rather than treated as continuous scores due to the nature of the distribution. Tertiles were created by allowing the statistical program to categorise the data based on its quantiles. For example, tertiles for some of the T4 variables were capped according to what would be considered a reasonable value for that factor, and cases were excluded if they exceeded these values. For instance, ‘time spent with other children/older children’ and ‘time spent free to move about’ were capped at 14 h per day as this has been reported as the mean waking time for child of this age [23]. ‘Time outdoors’ and ‘time spent being physically active with mum’ were capped at 7 h/day as this was considered to be half a waking day. In this way it was possible to assess child behaviours relative to other children in the sample.

Due to differences in child development, not all measures were relevant at each time-point, and hence were not assessed (for example, tummy time is no longer relevant once children begin crawling and walking and hence was not assessed beyond the 9 month time-point). Additionally, for some measures (maternal physical activity knowledge, parental facilitation of physical activity, and home equipment) the questions that comprised the measures differed between time-points. Two items included in the maternal physical activity knowledge scale at T1 (time 1), T2 (time 2) and T3 (time 3) were dropped at T4 (time 4) as they were no longer relevant due to the child’s age. Conversely, additional items (*n* = 4 at T3, *n* = 5 at T4) were added to the construct ‘parental facilitation of physical activity’ at T3 and T4 to more comprehensively assess this construct as children grew up. Finally, whilst the home environment score at T1-T3 was drawn from maternal reports of how ‘likely’ they were to provide equipment within the home over the coming year [22], at T4, the measure assessed how frequently the child used the piece of age-appropriate equipment at home. All constructs have shown adequate test-retest reliability in separate samples of infants, toddlers and preschool children [15, 22].

Children’s motor skill competence was assessed using the Test of Gross Motor Development- 2nd Edition (TGMD-2); considered a valid and reliable instrument [24]. It assesses 12 skills; six locomotor (run, gallop, hop, leap, horizontal jump, and slide) and six object control skills (striking a stationary ball, stationary dribble, kick, catch, overhand throw, and underhand roll), according to established protocols [24]. Motor skill data has been published previously for a subsample of the current sample who also had accelerometry data [25]. In brief, each skill was performed twice and video recorded in the home setting. If the home did not have the space in the backyard or front yard for the assessment, the garage space was sought or the front nature strip and in some cases local parks. Videos were coded by two experienced raters who had acceptable reliability on another study [26]. Inter-rater reliability using Intra Class Correlations (ICC) was assessed on a random subsample of 30 children at age 5 years for all 12 skills (ICC = 0.76, 95% CI 0.56, 0.88) [25] and for object control (ICC = 0.83, 95% CI 0.67, 0.92) and locomotor (ICC = 0.70, 95% CI 0.45, 0.84) skills. Both attempts of a skill were scored (‘1’ if component of the skill was correctly executed or ‘0’ if not) and summed. Relevant skills were summed for a total locomotor and total object control skill score.

Data were analysed using Stata v. 15.0. Proportions and means were derived as descriptive statistics and presented in Table 1. Separate multivariate linear regression models (eight in total) were used to examine the association between all available child and family related factors at each time-point and children’s locomotor and object control skills. In children aged 3 and older, correlates of skill differ according to how skill is operationalised and measured, so correlates were investigated separately in relation to object control and locomotor skills [7]. All models controlled for child age and sex (known covariates of motor skill competence [7]), the original intervention group and the cluster-based recruitment method. The statistical model at child aged 19 months also controlled for the age the child began walking, as it was associated with children’s physical activity levels in earlier analyses with this sample group [22].

## Results

Table 1 presents descriptive data for each of the predictors at each time-point either as means/standard deviations, proportions (% yes/no) or the tertile cut points. Table 2 describes the demographic characteristics of the study sample (mothers and children). There are almost even numbers of boys and girls in the sample. Mothers were mainly born in Australia, have a mean age of 32 and more than half have a University education. Compared to those lost to follow-up at 5 years old, the current sample was comprised of mothers who were more likely to have a university education and less likely to have a secondary school as their highest educational attainment (Chi^2^ = 5.5 *p* < 0.05). Children in the present sample were also slightly younger (Mean [SD] = 3.61 [0.99] vs. 4.12 [1.81] months at baseline).

**Table 2:** Demographic characteristics of Melbourne InFANT Program sample

Child characteristic	
Age in months [mean (SD)]	3.6 (0.99)
Sex (% male)	51.6%
Age in months child began crawling [mean (SD)]^a^	8.2 (1.7)
Age in months child began walking [mean (SD)]^a^	13.0 (1.70)
Maternal characteristic	
Age in years	32.4 (4.10)
Education	
Low (Secondary school or less)	17.6%
Medium (Trade certificate or diploma)	22.6%
High (University bachelor’s degree or higher)	59.8%
Country of birth	
Australia	83.2%
Other	16.8%

^a^Calculated from those children with complete data available for analysis at 19 months old

Table 3 reports associations between child and family factors in early life and children’s locomotor skill at 5 years old. At 4 months, maternal optimism was positively associated with locomotor skill score. At 9 months, physical activity equipment in the home was positively associated and maternal physical activity negatively associated with children’s locomotor skill score. Further, children in the middle and highest tertile for time outdoors had better locomotor skill score compared to those in the lowest tertile. Children who spent the most time (highest tertile) free to move about at 19 months and those children in the middle and highest tertile for time spent with older children at 3·5 years, had better locomotor skill score compared to the lowest tertile groups at each time-point. Lastly, children who spent the most time being physically active with mum at 3.5 years had lower locomotor skill scores.

**Table 3:** Child and family behaviours at child aged 4 months, 9 months, 19 months, 3.5 years and associations with locomotor skill at 5 years old^a^

	Predictors of locomotor skill (potential range 0–48) at each age			
Child and family behaviours	Child at 4 months (*n* = 256) β (95% CI)	Child at 9 months (*n* = 259) β (95% CI)	Child at 19 months (*n* = 195)^b^ β (95% CI)	Child at 3.5 years (*n* = 178) β (95% CI)
Child behaviours				
Time spent being physically active with mum				
Lowest tertile	Ref.	Ref.	Ref.	Ref.
Middle tertile	−0.89 (− 3.06, 1.27)	0.26 (− 1.96, 2.47)	− 2.27 (−4.72, 0.16)	− 1.12 (− 3.52, 1.30)
Highest tertile	− 1.68 (− 4.46, 1.10)	−1.66 (− 4.19, 0.87)	−1.36 (− 4.28, 1.57)	−**3.73 (−6.68, − 0.78)**
Time spent having tummy time				
Lowest tertile	Ref.	N/A	N/A	N/A
Middle tertile	0.19 (−1.93 (2.31)	–	–	–
Highest tertile	1.75 (− 0.61, 4.12)	–	–	–
Time spent on the floor/free to move about^c^				
Lowest tertile	Ref.	Ref.	Ref.	Ref.
Middle tertile	0.37 (−1.92, 2.65)	−0.13 (−2.37 (2.10)	1.07 (− 1.30, 3.44)	1.98 (− 0.64, 4.60)
Highest tertile	− 0.60 (−3.17, 1.96)	0.29 (− 2.15, 2.73)	**2.41 (0.02, 4.80)**	− 1.30 (− 3.84, 1.22)
Time spent with other children of a similar age				
Lowest tertile	Ref.	Ref.	Ref.	Ref.
Middle tertile	0.63 (−1.47, 2.74)	−1.52 (−3.62, 0.56)	0.38 (− 2.20, 2.97)	−1.16 (− 3.71, 1.38)
Highest tertile	0.90 (− 1.33, 3.14)	− 1.53 (− 3.98, 0.91)	− 0.43 (− 2.90, 2.03)	0.04 (− 2.74, 2.83)
Time spent with older children				
Lowest tertile	Ref.	Ref.	Ref.	Ref.
Middle tertile	1.37 (−0.64, 3.38)	− 0.00 (− 2.18, 2.18)	1.31 (− 1.57, 4.21)	**3.15 (0.72, 5.59)**
Highest tertile	0.35 (− 1.82, 2.53)	− 0.41 (− 2.66, 1.84)	0.21 (−2.23, 2.66)	**3.00 (0.09, 5.91)**
Time spent outside				
Lowest tertile	Ref.	Ref.	Ref.	Ref.
Middle tertile	0.30 (− 1.90, 2.50)	**2.50 (0.39, 4.62)**	0.59 (−1.89, 3.07)	1.82 (−0.90, 4.54)
Highest tertile	0.68 (−1.51, 2.88)	**2.86 (0.47, 5.26)**	1.44 (− 1.26, 4.13)	1.65 (−1.21, 4.51)
Organised activity (Ref. No)	N/A	N/A	Ref.	Ref.
Yes	–	**–**	−1.19 (− 3.23, 0.84)	− 1.67 (− 0.94, 4.29)
Maternal beliefs				
Maternal physical activity knowledge	−2.37 (−5.15, 0.41)	N/A	−1.54 (−4.73, 1.65)	− 2.21 (−5.57, 1.15)
Maternal physical activity views	1.54 (− 0.80, 3.88)	N/A	0.88 (− 1.83, 3.59)	0.61 (− 2.06, 3.28)
Maternal physical activity optimism	**2.43 (0.12, 4.75)**	−0.62 (− 2.94, 1.70)	− 0.40 (− 2.80, 2.01)	1.00 (− 1.48, 3.47)
Maternal physical activity self-efficacy	− 1.03 (− 3.24, 1.18)	0.42 (− 1.69, 2.54)	2.16 (− 0.32, 4.65)	1.61 (− 0.88, 4.09)
Maternal floor concerns	− 0.31 (− 2.04, 1.43)	N/A	N/A	N/A
Parental behaviours				
Parental facilitation of physical activity (mins/week)	0.06 (− 0.11, 0.24)	0.02 (− 0.15, 0.19)	− 0.02 (− 0.12, 0.07)	0.01 (− 0.11, 0.11)
Maternal physical activity (mins/week)	− 0.00 (− 0.00, 0.00)	**−0.01 (− 0.01, − 0.00)**	−0.00 (− 0.00, 0.00)	−0.00 (− 0.00, 0.01)
Home environment				
Physical activity equipment in home	0.15 (−0.27, 0.57)	**0.82 (0.05, 1.59)**	0.29 (−0.23, 0.81)	0.06 (− 0.07, 0.19)

^a^All models controlled for intervention group, sex and age of child and clustering by parents group attended. N/A = not assessed at particular time point

^b^Also controlled for age child began walking

^c^At 19 months and 3.5 years old, this variable was termed ‘free to move about’ rather than ‘on the floor’

Bolded results are significant at *p* < 0.05

Table 4 reports the associations between child and family factors at 4, 9, 19 months and 3.5 years and children’s object control skill at 5 years. At 4 months, those in the highest tertile for time spent with older children had higher object control skill score compared to those in the lowest tertile. At 19 months, those in the middle and highest tertiles for time spent free to move about and those in the middle tertile for time spent with older children, had better object control skills compared to those in the lowest tertile. At both 9 months and 3.5 years, children with more equipment in the home had higher object control skills. Maternal physical activity knowledge at 3.5 years was negatively associated with children’s object control skill.

**Table 4:** Child and family behaviours at child aged 4 months, 9 months, 19 months, 3.5 years and associations with object control skill at 5 years old^a^

	Predictors of object control skill (potential range 0–48) at each age			
Child and family behaviours	Child at 4 months (*n* = 256) β (95% CI)	Child at 9 months (*n* = 259) β (95% CI)	Child at 19 months (*n* = 195)^b^ β (95% CI)	Child at 3.5 years (*n* = 178) β (95% CI)
Child behaviours				
Time spent being physically active with mum				
Lowest tertile	Ref.	Ref.	Ref.	Ref.
Middle tertile	0.15 (− 1.89, 2.20)	0.44 (− 1.49, 2.37)	−0.50 (− 2.78, 1.76)	0.83 (− 2.89, 1.23)
Highest tertile	−1.94 (− 4.56, 0.69)	−1.44 (− 3.64, 0.77)	−1.49 (− 4.22, 1.23)	−1.30 (− 3.89, 1.13)
Time spent having tummy time				
Lowest tertile	Ref.	N/A	N/A	N/A
Middle tertile	0.81 (−1.20, 2.81)	–	–	–
Highest tertile	1.71 (−0.52, 3.94)	–	–	–
Time spent on the floor^c^				
Lowest tertile	Ref.	Ref.	Ref.	Ref.
Middle tertile	−0.48 (− 2.64, 1.68)	0.25 (−1.71, 2.19)	**2.55 (0.34, 4.76)**	−0.91 (− 3.12, 1.31)
Highest tertile	0.98 (−1.45, 3.41)	0.02 (− 2.10, 2.14)	**2.47 (0.25, 4.70)**	−2.14 (− 4.29, 0.01)
Time spent with other children of a similar age				
Lowest tertile	Ref.	Ref.	Ref.	Ref.
Middle tertile	0.06 (−1.93, 2.06)	−0.70 (−2.53, 1.12)	− 0.87 (− 3.27, 1.53)	1.60 (− 0.57, 3.77)
Highest tertile	0.69 (− 1.43, 2.80)	− 0.54 (− 2.68, 1.60)	0.11 (− 2.18, 2.41)	1.31 (− 1.07, 3.67)
Time spent with older children				
Lowest tertile	Ref.	Ref.	Ref.	Ref.
Middle tertile	1.35 (−0.55, 3.26)	0.32 (−1.58, 2.22)	**2.97 (0.28, 5.66)**	0.07 (−1.99, 2.13)
Highest tertile	**2.27 (0.21, 4.33)**	1.45 (−0.51, 3.41)	0.73 (−1.55, 3.01)	−0.28 (−2.74, 2.19)
Time spent outside				
Lowest tertile	Ref.	Ref.	Ref.	Ref.
Middle tertile	−0.54 (−2.62, 1.54)	1.03 (− 0.82, 2.88)	0.12 (− 2.20, 2.42)	−0.30 (− 2.60, 1.99)
Highest tertile	− 0.90 (− 2.98, 1.8)	1.30 (− 0.79, 3.40)	0.33 (− 2.18, 2.84)	2.26 (− 0.17, 4.70)
Organised activity (Ref. No)	N/A	N/A	Ref.	Ref.
Yes	–	**–**	−0.51 (− 2.41, 1.38)	1.11 (−1.07, 3.29)
Maternal beliefs				
Maternal physical activity knowledge	−0.68 (−3.31, 1.94)	N/A	−0.83 (− 3.80, 2.13)	**−3.05 (−5.91, − 0.19)**
Maternal physical activity views	−0.69 (− 2.90, 1.52)	N/A	0.41 (− 2.11, 2.93)	− 0.15 (− 2.43, 2.12)
Maternal physical activity optimism	−0.88 (− 3.07, 1.30)	−0.51 (− 2.54, 1.51)	−0.86 (− 3.09, 1.39)	0.23 (− 1.89, 2.34)
Maternal physical activity self-efficacy	−0.39 (− 2.48, 1.70)	−0.44 (− 2.28, 1.41)	0.73 (− 1.59, 3.05)	0.73 (− 1.38, 2.84)
Maternal floor concerns	0.19 (− 1.45, 1.83)	N/A	N/A	N/A
Parental behaviours				
Parental facilitation of physical activity (mins/week)	0.01 (−0.15, 0.18)	−0.02 (− 0.16, 0.13)	−0.03 (− 0.12, 0.06)	−0.02 (− 0.11, 0.08)
Maternal physical activity (mins/week)	0.00 (− 0.00, 0.01)	0.00 (− 0.00, 0.00)	0.00 (− 0.00, 0.00)	−0.00 (− 0.00, 0.00)
Home environment				
Physical activity equipment in home	0.26 (−0.13, 0.66)	**0.83 (0.16, 1.51)**	0.21 (−0.28, 0.70)	**0.17 (0.06, 0.28)**

^a^All models controlled for intervention group, sex and age of child and clustering by parents group attended. N/A = not assessed at particular time point

^b^Also controlled for age child began walking

^c^At 19 months and 3.5 years old, this variable was termed ‘free to move about’ rather than ‘on the floor’

Bolded results are significant at *p* < 0.05

## Discussion

The purpose of this study was to investigate a range of potentially modifiable infant, toddler and early preschool child, family and home environment factors hypothesised to predict children’s motor competence at age 5 years. This is the first such study to investigate early life correlates of motor competence longitudinally from infancy through to 5 years.

Generally, the study showed that the home environment had some positive effects on their child’s subsequent motor skill development. Some of the stronger associations showed that an additional three points in skill could be achieved through certain environmental elements. This can be interpreted as three additional skill components correct in one trial of a skill (skills have between 3 and 5 components to be mastered). While we might expect stronger associations for the more proximal time-points, or that certain earlier experiences might be more important for subsequent motor skill development, overall, there were not clear patterns in the associations. Instead, there were a variable number of factors associated with both skill types at the different age points.

The most consistent association observed was for the number of age-appropriate toys and equipment available in the home environment. This was important at 9 months of age for locomotor and object control skills where there was an average of 6 home based toys suitable for physical activity. At 3.5 years this item changed to assess how frequently children used the age-appropriate physical activity equipment items on a scale of never to daily. Engaging in toy use daily (on average) in 3.5 year olds was also associated with object control skills at 5 years old. It is logical that available toys and equipment could help to stimulate both locomotor and object control skills, as this survey question could include any sort of toy relevant to physical activity. This finding confirms a recent study in American preschool children which reported that physical activity equipment and/or play spaces present in the home was positively related with locomotor skills [16] and replicates other cross-sectional studies in infants [10, 11, 13] and preschool children [15]. In apparent contrast, one previous study found that the frequency of buying equipment/toys was not associated with preschool children’s motor skills [14], although it is reasonable that the *number* of toys/equipment and the frequency of *use* of such toys relate better to motor skill than the *frequency* with which they are bought.

At 19 months, children in the top tertile of time spent freely moving about (i.e. responses between 510 and 1440 min per week - approximately 73 min per day to 206 min per day), had significantly higher object and locomotor skills. Our finding supports the Australian 24-Hour Movement Guidelines for the Early Years, which state that toddlers (1 to 2 years) should be encouraged to “move regularly throughout the day, and not be restrained for long periods” [27]. This relationship wasn’t apparent at the 3.5 year old time-point which could be because children were generally much less restrained at this point. Interestingly, this relationship was not yet apparent in infancy, despite floor-based play being identified in guidelines as important at such ages [27]. It is possible that in this study children were not engaged in the types of activities that are necessary to positively impact motor skills when placed on the floor as infants, as only total floor time was assessed. Alternatively, it is possible that the benefits of floor based play in infancy are not strong enough to be independently associated with motor competence at 5 years of age.

Time spent outside at 9 months was predictive of locomotor skill with children who spent between 34 and 69 min per week (mid tertile) and children who spent 77–257 minnutes per week outside (high tertile) having better skills. Outdoor spaces and the novelty they may provide, might encourage locomotor development (crawling, scooting) in infants. It may also reflect that the child had more space to move around and that parental practices were less restrictive. It was surprising that outdoor time at preschool age did not relate to locomotor skills, given physical activity is correlated with motor skills and a recent systematic review in 3–12 year olds found outdoor time was consistently associated with more physical activity and less sedentary behaviour compared to indoor time [28]. An older cross-sectional Finnish study in children aged 3–4 years, showed that parent-reported time playing outdoors was associated with locomotor skill in terms of faster running, although, other skills (both object control and locomotor) were not associated with outdoor play [29]. Therefore, whilst it appears that time outside benefits physical activity, the potential for specific motor skill benefits may be less clear. Although, a recent representative study in Finnish preschool aged children showed time outdoors was related to both object control and locomotor skills [30]. A limitation of the current study is that physical activity type was not assessed. Identifying what activities children engage in when they are outdoors (or indoors) at different ages may help determine optimal activity types as well as duration of outdoor (or indoor) activity needed for development of which types of motor competence.

Time spent with older children was associated at 4 months (for children spending between 26 and 343 min per week – high tertile) and 19 months of age (10–30 min per week – medium tertile), and at 3·5 years (50–420 min per week – medium tertile), with object control and locomotor skills respectively. From a motor development perspective this could be due to observation and imitation, i.e., a study in preschool aged siblings (5·4 and 3·5 years), found that older children usually initiated body-oriented tasks (e.g. crawl, walk, climb, jump) whilst the younger siblings watched, and that younger children often then imitated the tasks their older siblings had completed [31]. In physical activity research, it has been demonstrated in 3 to 6 year olds that being with another child facilitated more physical activity than when alone [32] and in older children (6–11 years), having siblings in the home was highly associated with physical activity [33]. It is unclear why the highest tertile was not associated with either object control or locomotor skills at 19 months and 3.5 years, perhaps there is a ceiling effect in terms of the benefit a child can gain when playing with an older child.

Interestingly, maternal optimism about physical activity when their baby was 4 months was important to subsequent locomotor skill (but not object control skill). It is also important to discuss the factors that were not associated with either skill type. In terms of child factors, time spent having tummy time at 4 months was not associated with either subsequent object control or locomotor skills. This may be because our measure of motor competence at age 5 years did not include a specific measure of stability. Previous research has demonstrated that motor object control and locomotor skills are separate constructs to stability [34]. Tummy time is considered important for infant development, particularly for reducing the severity of deformational plagiocephaly (skull deformity from repeated pressure due to head being in one position) [35]. Another child factor with no association, was organised activity, which has been reported as a predictor of motor competence when children are preschool aged [15]. We investigated it because many parents enrol their children in learn to swim classes and baby gym, which might be expected to influence subsequent motor skills. The inconsistency in results in the current study may be due to sample characteristics (i.e. only one fifth of our sample did no organised activity) or the nature of the organised activities that children engaged in. For example, children may have been engaged in music classes or other activities that don’t have a specific obvious benefit to gross motor skill development.

In terms of family levels factors, with no associations or surprising associations, maternal self-reported physical activity at child aged 9 months was negatively associated with locomotor skill and thus was not in the hypothesised direction; however, this relationship was negligible. Time being active with mum for 3.5 year olds also had a negative relationship with locomotor skills. Potentially this could be due to how a parent defined as active time. If they were jogging/walking whilst child was restrained this would negatively affect skill development for instance. Parent facilitation of physical activity was not associated with either object control or locomotor skill at any time point which is a surprising finding. Perhaps parents views of what they do regarding physical activity facilitation does not tie closely with motor development, for instance they might provide unstructured playtime which in intervention trials has been shown to not improve children’s motor competence when compared to structured interventions [36].

The study strengths include the longitudinal nature of the unique dataset with multiple time points across early childhood, the sample size, the reliable assessment of motor skills and the analytical methods adjusting for potential confounding variables and clustering. A challenge for analysis was that questions had to reflect the children’s growth and development so they changed at each time-point. It is important to note that behaviours are a best estimate by parents and thus can be treated as an indicator rather than being able to determine definitive times in certain behaviours and their relationship with motor skill. A high proportion of the sample had educated parents thus affecting the generalisability of the results. Future research may wish to consider gaining further information regarding other key significant others, in particular fathers, as fathers physical activity has been associated with 4–6 year old children’s skills in a prior study [14].

## Conclusion

In conclusion, it is important to advise parents that the home environment can make a difference to their child’s motor competence and they can start right from infancy. Providing a home space with toys and equipment to facilitate physical activity, maximising time for toddlers to be free to move about, providing opportunities for their toddler and preschool child to play with older children (such as play group and kinder), and time outside, are all factors that will assist children to develop the skills they need to be physically active. These specific activities could also help inform physical activity guidelines for parents of young children. Whilst our findings support principles of the 24-Hour Movement Guidelines for the Early Years, they also emphasise the importance of the physical activity context to parents and caregivers, i.e. encouraging time for your child to play with others, be outside and have the equipment to facilitate physical activity and subsequent motor development. Thus, it is recommended that the context of physical activity be specified in future guidelines to help clinicians and parents understand what they can do to enhance children’s motor development.

What is also clear from this study is that there were large temporal variations in significant associations across the different time points and between the skill domains. Potentially the changing contexts over time and developmental milestones as they age may have contributed to this mixed picture. Future research may seek to investigate these factors more closely so we can work to better understand how best to ensure children have the motor competence they need to be physically active.

## Data Availability

The datasets used and/or analysed during the current study are available from the corresponding author on reasonable request.

## References

[1] Robinson LE, Stodden DF, Barnett LM, Lopes VP, Logan SW, Rodrigues LP (2015). Motor Competence and its Effect on Positive Developmental Trajectories of Health. Sports Med (Auckland, NZ).

[2] Cattuzzo MT, Dos Santos HR, Re AH, de Oliveira IS, Melo BM, de Sousa MM (2016). Motor competence and health related physical fitness in youth: a systematic review. J Sci Med Sport.

[3] Hardy LL, Barnett LM, Espinel P, Okely AD (2013). Thirteen-year trends in child and adolescent fundamental movement skills: 1997–2010. Med Sci Sports Exerc.

[4] O’Brien W, Belton S, Issartel J (2016). Fundamental movement skill proficiency amongst adolescent youth. Phys Educ Sport Pedagog.

[5] Valentini NC, Logan SW, Spessato BC, MSd S, Pereira KG, Rudisill ME (2016). Fundamental motor skills across childhood: age, sex, and competence outcomes of Brazilian children. J Motor Learn Dev.

[6] Engel AC, Broderick CR, van Doorn N, Parmenter BJ, Hardy LL (2018). Exploring the relationship between fundamental motor skill interventions and physical activity levels in children: a systematic review and meta-analysis. Sports Med.

[7] Barnett LM, Lai SK, Veldman SLC, Hardy LL, Cliff DP, Morgan PJ (2016). Correlates of gross motor competence in children and adolescents: a systematic review and meta-analysis. Sports Med.

[8] Iivonen S, Sääkslahti AK (2013). Preschool children's fundamental motor skills: a review of significant determinants. Early Child Dev Care.

[9] Baxter JA, Hand K, Sweid R (2016). Flexible child care and Australian parents’ work and care decision-making (Research Report No. 37).

[10] Cacola P, Gabbard C, Santos DC, Batistela AC (2011). Development of the affordances in the home environment for motor development-infant scale. Pediatr Int.

[11] Mori S, Nakamoto H, Mizuochi H, Ikudome S, Gabbard C (2013). Influence of affordances in the home environment on motor development of young children in Japan. Child Dev Res.

[12] Miquelote AF, Santos DCC, Caçola PM (2012). Montebelo MIdL, Gabbard C. effect of the home environment on motor and cognitive behavior of infants. Infant Behav Dev.

[13] Prado EL, Souheila A, Seth AA, Mary A, Per A, Ulla A (2017). Predictors and pathways of language and motor development in four prospective cohorts of young children in Ghana, Malawi, and Burkina Faso. J Child Psychol Psychiatry.

[14] Cools W, Martelaer K (2011). D., Samaey C, Andries C. fundamental movement skill performance of preschool children in relation to family context. J Sports Sci.

[15] Barnett LM, Hinkley T, Okely AD, Salmon J (2013). Child, family and environmental correlates of children’s motor skill proficiency. J Sci Med Sport.

[16] Zeng N, Johnson SL, Boles RE, Bellows LL (2019). Social-ecological correlates of fundamental movement skills in young children. J Sport Health Sci.

[17] Valla L, Birkeland MS, Hofoss D, Slinning K (2017). Developmental pathways in infants from 4 to 24 months. Child Care Health Dev.

[18] Neelon SEB, Oken E, Taveras EM, Rifas-Shiman SL, Gillman MW (2012). Age of achievement of gross motor milestones in infancy and adiposity at age 3 years. Matern Child Health J.

[19] Ghassabian A, Sundaram R, Bell E, Bello SC, Kus C, Yeung E (2016). Gross Motor Milestones and Subsequent Development. Pediatrics.

[20] Pereira KR, Valentini NC, Saccani R (2016). Brazilian infant motor and cognitive development: longitudinal influence of risk factors. Pediatr Int.

[21] Campbell K, Hesketh K, Crawford D, Salmon J, Ball K, McCallum Z (2008). The infant feeding activity and nutrition trial (INFANT) an early intervention to prevent childhood obesity: cluster-randomised controlled trial. BMC Public Health.

[22] Hnatiuk J, Salmon J, Campbell K, Ridgers N, Hesketh K (2013). Early childhood predictors of toddlers’ physical activity: longitudinal findings from the Melbourne InFANT program. Int J Behav Nutr Phys Act.

[23] Iglowstein I, Jenni OG, Molinari L, Largo RH (2003). Sleep duration from infancy to adolescence: reference values and generational trends. Pediatrics.

[24] Ulrich DA. Test of Gross Motor Development (2nd ed). Austin, TX 2000. PRO-ED

[25] Barnett LM, Salmon J, Hesketh KD (2016). More active pre-school children have better motor competence at school starting age: an observational cohort study. BMC Public Health.

[26] Barnett LM, Minto C, Lander N, Hardy LL (2014). Interrater reliability assessment using the test of gross motor Development-2. J Sci Med Sport.

[27] Australian Government Department of Health (2017). Australian 24-Hour Movement Guidelines for the Early Years (Birth to 5 years): An Integration of Physical Activity, Sedentary Behaviour, and Sleep.

[28] Gray C, Gibbons R, Larouche R, Sandseter E, Bienenstock A, Brussoni M (2015). What is the relationship between outdoor time and physical activity, sedentary behaviour, and physical fitness in children? A systematic review. Int J Environ Res Public Health.

[29] Sääkslahti A, Numminen P, Niinikoski H, Rask-Nissilä L, Viikari J, Tuominen J (1999). Is physical activity related to body size, fundamental motor skills, and CHD risk factors in early childhood?. Pediatr Exerc Sci.

[30] Niemistö D, Finni T, Haapala EA, Cantell M, Korhonen E, Sääkslahti A (2019). Environmental correlates of motor competence in children—the skilled kids study. Int J Environ Res Public Health.

[31] Erbaugh SJ, Clifton MA (1984). Sibling relationships of preschool-aged children in gross motor environments. Res Q Exerc Sport.

[32] Barkley JE, Salvy S-J, Sanders GJ, Dey S, Carlowitz K-PV, Williamson ML (2014). Peer influence and physical activity behavior in young children: an experimental study. J Phys Act Health.

[33] Tandon P, Grow HM, Couch S, Glanz K, Sallis JF, Frank LD (2014). Physical and social home environment in relation to children’s overall and home-based physical activity and sedentary time. Prev Med.

[34] Rudd JR, Barnett LM, Butson ML, Farrow D, Berry J, Polman RCJ (2015). Fundamental movement skills are more than run, throw and catch: the role of stability skills. PLoS One.

[35] Wittmeier K, Mulder K (2017). Time to revisit tummy time: a commentary on plagiocephaly and development. Paediatr Child Health.

[36] Logan SW, Robinson LE, Wilson AE, Lucas WA (2012). Getting the fundamentals of movement: a meta-analysis of the effectiveness of motor skill interventions in children. Child Care Health Dev.

